# A strong association between non-musculoskeletal symptoms and musculoskeletal pain symptoms: results from a population study

**DOI:** 10.1186/1471-2474-12-285

**Published:** 2011-12-18

**Authors:** Hedda Tschudi-Madsen, Mona Kjeldsberg, Bård Natvig, Camilla Ihlebaek, Ingvild Dalen, Yusman Kamaleri, Jørund Straand, Dag Bruusgaard

**Affiliations:** 1 Department of General Practice, Institute of Health and Society, Faculty of Medicine, University of Oslo, PO BOX 1130, Blindern, 0318 Oslo, Norway; 2 Department of Community Health, Institute of Health and Society, Faculty of Medicine, University of Oslo, Oslo, Norway; 3 National Resource Centre for Rehabilitation in Rheumatology, Diakonhjemmet Hospital, Oslo, Norway; 4 Health UMB, IHA, University of Life Sciences (UMB), Aas, Norway; 5 Uni Health, Norway; 6 Department of Rare Diseases and Disabilities, Oslo University Hospital Ullevål, Oslo, Norway

**Keywords:** Epidemiology, Cross-sectional, General population, Musculoskeletal pain, Medically unexplained symptoms

## Abstract

**Background:**

There is a lack of knowledge about the pattern of symptom reporting in the general population as most research focuses on specific diseases or symptoms. The number of musculoskeletal pain sites is a strong predictor for disability pensioning and, hence, is considered to be an important dimension in symptom reporting. The simple method of counting symptoms might also be applicable to non-musculoskeletal symptoms, rendering further dimensions in describing individual and public health. In a general population, we aimed to explore the association between self-reported non-musculoskeletal symptoms and the number of pain sites.

**Methods:**

With a cross-sectional design, the Standardised Nordic Questionnaire and the Subjective Health Complaints Inventory were used to record pain at ten different body sites and 13 non-musculoskeletal symptoms, respectively, among seven age groups in Ullensaker, Norway (n = 3,227).

**Results:**

Results showed a strong, almost linear relationship between the number of non-musculoskeletal symptoms and the number of pain sites (r = 0.55). The *number *and *type *of non-musculoskeletal symptoms had an almost equal explanatory power in the number of pain sites reported (27.1% vs. 28.2%).

**Conclusion:**

The linear association between the number of non-musculoskeletal and musculoskeletal symptoms might indicate that the symptoms share common characteristics and even common underlying causal factors. The total burden of symptoms as determined by the number of symptoms reported might be an interesting generic indicator of health and well-being, as well as present and future functioning. Research on symptom reporting might also be an alternative pathway to describe and, possibly, understand the medically unexplained multisymptom conditions.

## Background

Patients present to their general practitioners (GPs) with symptoms, not a diagnosis. Despite this, symptomatology is traditionally viewed in the context of predefined illness. Most research focuses on a particular disease or on selected symptoms. Knowledge about the pattern of symptom reporting may be of importance to decision making in general practice; it may affect referral patterns and the tendency to initiate further tests.

In a recent article describing the symptom iceberg in the UK population, prevalence figures for common symptoms were presented in relation to individual characteristics and chronic conditions [1]. In a review, Kroenke has shown that 80% of individuals in a general population will experience at least one symptom during a given month [2]. Furthermore, he states that at least one third of the symptoms reported in primary care and in population-based studies are "medically unexplained", ranging from 20% to75% depending on methods used for classifying symptoms.

Symptoms for which there is no evident medical explanation pose a special challenge to the health care system in general, to GPs in particular, as well as to society as a whole due to costs related to utilisation of health care and welfare resources[2-4]. Several terms have been launched to describe symptoms without plausible medical explanations [5,6]. "Medically unexplained symptoms" (MUS) is one of the most recent terms, although consensus regarding its applicability remains to be established [7]. Research in this field has traditionally focused on the individual syndromes. While a GP will face all syndromes, other clinical specialties seem to have their own "medically unexplained syndrome", such as fibromyalgia (rheumatology), chronic fatigue syndrome (neurology, infectious medicine), and irritable bowel disease (gastroenterology) [8]. Many researchers have pointed out the substantial symptom overlap between these syndromes, which implies that many patients meet the criteria listed for several of the syndromes [9-14].

Results from the Ullensaker study on self-reported musculoskeletal pain have shown that reporting a single pain site or none at all is rare, and almost two out of five individuals reported pain from at least five out of ten listed pain sites during the last year [15]^. ^Furthermore, a strong and linear relationship between the number of pain sites and functional ability has been described [16]. The number of pain sites is also a strong predictor for disability pensioning 14 years later [17]. It is well known that some individuals with musculoskeletal symptoms also report other common symptoms. For example, the non-musculoskeletal symptoms are incorporated in the new preliminary diagnostic criteria for fibromyalgia [18,19]. Furthermore, longitudinal studies in the UK have shown an association between persistence of chronic widespread pain and the reporting of other somatic symptoms reported in assessment of somatisation disorder [20-22].

When analysing symptoms presented in medical encounters, physicians need more insight into symptom reporting as a phenomenon in itself and as background knowledge. As a counterweight to research focusing on further subdivision of existing syndromes, one approach is to examine the reporting of common symptoms in a general population. Symptom reporting in the population might also be a way of describing and possibly understanding medically unexplained symptoms and syndromes.

The association between musculoskeletal pain and the continuum of other common symptoms, regardless of their innate nature, remains to be explored. This article aims to explore the association between self-reported non-musculoskeletal symptoms and musculoskeletal pain.

## Methods

### Study design and sample

This article is based on data from the Ullensaker Study, a cohort study focusing primarily on the epidemiology of musculoskeletal pain. Ullensaker is a suburban municipality, 40 km northeast of Oslo, Norway. Data for this study were collected in 2004 by sending a self-administered questionnaire to all inhabitants in the seven age groups: 24-26, 34-36, 44-46, 54-56, 64-66, 74-76, and 84-86 year-olds. A reminder was sent to non-responders after 6 weeks.

The Ullensaker study was approved by the Regional Committee of Research Ethics in Norway.

### Variables

We used the validated Standardised Nordic Questionnaire (SNQ) [23] to record musculoskeletal symptoms. Respondents were asked to report whether they had experienced pain or discomfort in any of ten different body regions during the last 7 days: head, neck, shoulder, elbow, hand/wrist, upper back, lower back, hip, knee, and ankle/foot. Response categories were restricted to 'no' and 'yes'. A body manikin was supplied to illustrate the location of the body regions. We constructed a simple sum score by counting the number of musculoskeletal pain sites (NPS), ranging from 0 to 10.

Non-musculoskeletal symptoms were a selection of 13 of the 29 items included in the validated Subjective Health Complaints Inventory (SCH) [24], which were not covered by the SNQ. Respondents were to report whether or not they had experienced any of the following complaints during the last 30 days: palpitations/extra heartbeats, chest pain, breathing difficulties, heart burn, stomach discomfort, diarrhoea, constipation, eczema, tiredness, dizziness, anxiety, depression, and sleep problems. For each item, four response categories were available: not at all, a little, some, and severe. During analyses, the answers were dichotomized into 'not at all' (code 0) vs. the rest (code 1), allowing an overall sum score of the number of non-musculoskeletal symptoms (NN-MS, ranging from 0 to 13).

We also constructed a sum score for the total number of symptoms reported by adding NN-MS and NPS scores, ranging from 0 to 23.

### Statistical analyses

We performed the following imputation procedures. A number of respondents only ticked "yes" or "no" for some pain sites (15.3%) and other symptoms (9.0%), and did not tick for the rest. For blank answers we assumed that the symptom/pain was not present and they were consequently coded as "not present". Imputations were done for a total of 21.2% of the respondents. To control for how these imputation procedures might influence the results, we performed sensitivity analyses where all analyses were performed on non-imputated data.

Frequencies and percentages were used to describe the prevalence of NPS and NN-MS, and Pearson's correlation analysis was used to describe the correlation between the two scores.

The following linear regression analyses were performed after checking for multicollinearity between symptoms, all using NPS as the dependent variable. Model I assessed the explanatory power of NN-MS, while controlling for age and gender. In model II NPS was seen as a function of all the 13 non-musculoskeletal symptoms individually, adjusted for age and gender. In model III, the 13 non-musculoskeletal symptoms were modelled individually, controlling for the remaining 12 symptoms in addition to age and gender.

Unstandardised regression coefficients (β- values) are presented, with 95% confidence intervals (obtained using Agresti-Coull intervals from an online calculator) and multiple correlation coefficients, R^2^.We also performed regression analyses assessing the explanatory power of NN-MS in NPS when including only non-musculoskeletal symptoms affecting the respondent to "some" and "severe" degree, and to "severe" degree, respectively.

All analyses were performed using SPSS for Windows (version 16).

## Results

### Study sample

The questionnaire was sent to 6,105 persons, and after one reminder, 3,325 individuals responded, giving a response rate of 54.4%. Individuals who had not answered any of the questions on pain and those who had not answered any questions on non-musculoskeletal symptoms were excluded (n = 98), resulting in a final sample of 3,227 individuals (52.9% of the original sample size). The participation rate was higher in women (59%) than men (49%), and higher in middle-aged groups for both genders [14]. Of respondents, 54.9% were women and 45.1% men. The distribution of respondents within age groups was as follows (the percentage within each age group in our census population in parentheses[25]): 24-26: 9.8% (16.5%), 34-36: 29.2% (25.2%), 44-46: 18.5% (18.7%), 54-56: 20.6% (17.8%), 64-66: 13.5% (11.0%), 74-76: 7.0% (7.4%), and 84-86 year olds: 1.5% (3.2%). Accordingly, non-responders are mostly to be found among the youngest and oldest age groups.

### The number of symptoms

The respondents reported a mean of 2.3 pain sites (95% CI 2.2-2.4), women 2.8 (95% CI 2.6-2.9), and men 1.8 (95% CI 1.7-1.9). Table 1 shows the prevalence of NPS.

**Table 1:** Prevalence of number of pain sites (NPS) by gender

NPS	Men (N = 1455)	Women (N = 1772)	Total (N = 3227)
	%(95%CI)	%(95%CI)	%(95%CI)
**0**	38.4 (36.2 to 41.2)	23.2 (21.3 to 25.2)	30.1 (28.5 to 31.7)

**1**	18.2 (16.3 to 20.3)	15.7 (14.1 to 17.5)	16.8 (15.5 to 18.1)

**2**	12.4 (10.8 to 14.2)	14.6 (13.0 to 16.3)	13.5 (12.4 to 14.8)

**3**	12.8 (11.2 to 14.6)	13.0 (11.6 to 14.7)	12.9 (11.8 to 14.1)

**4**	7.7 (6.4 to 9.2)	10.7 (9.31 to 12.2)	9.3 (8.3 to 10.4)

**5**	4.5 (3.5 to 5.7)	7.7 (6.5 to 9.0)	6.2 (5.4 to 7.1)

**6**	3.0 (2.3 to 4.1)	5.6 (4.6 to 6.8)	4.4 (3.8 to 5.2)

**7**	1.2 (0.7 to 1.9)	4.2 (3.3 to 5.2)	2.8 (2.3 to 3.5)

**8**	0.7 (0.4 to 1.3)	2.1 (1.6 to 2.9)	1.5 (1.1 to 2.0)

**9**	0.8 (0.4 to 1.4)	1.7 (1.2 to 2.4)	1.3 (0.9 to 1.7)

**10**	0.8 (0.4-1.4)	1.6 (1.1 to 2.3)	1.2 (0.9 to 1.7)

NPS Number of pain sites: CI Confidence Interval

Table 2 shows the prevalence of NN-MS.

**Table 2:** Prevalence of number of non-muscular symptoms (NN-MS) by gender

NN-MS	Men (N = 1455)	Women (N = 1772)	Total (N = 3227)
	% (95% CI)	% (95% CI)	% (95% CI)
**0**	16.8 (15.0 to 18.8)	9.9 (8.6 to 11.4)	13.0 (11.9 to 14.2)

**1**	14.7(13.0 to 16.7)	12.4 (11.0 to 14.0)	13.4 (12.3 to 14.6)

**2**	14.1 (12.4 to 16.0)	15.1 (13.5-16.9)	14.6 (13.5 to 15.9)

**3**	13.6 (11.9 to 15.4)	11.9 (10.4 to 13.4)	12.6 (11.5 to 13.8)

**4**	12.0 (10.5 to 13.8)	11.6 (10.2 to 13.2)	11.8 (10.7 to 13.0)

**5**	8.7 (7.4 to 10.3)	11.1 (9.7 to 12.7)	10.0 (9.0 to 11.1)

**6**	7.1 (5.8 to 8.5)	7.6 (6.4 to 8.9)	7.3 (6.5 to 8.3)

**7**	5.8 (4.7 to 7.2)	7.5 (6.4 to 8.8)	6.7 (5.9 to 7.6)

**8**	2.8 (2.0 to 3.8)	5.1 (4.2 to 6.2)	4.0 (3.4 to 4.8)

**9**	2.1 (1.5 to 3.0)	3.1 (2.3 to 4.0)	2.6 (2.1 to 3.2)

**10**	1.7 (1.2 to 2.6)	2.3 (1.7 to 3.1)	2.1 (1.6 to 2.6)

**11**	0.6 (0.3 to 1.1)	1.5 (1.0 to 2.2)	1.1 (0.8 to 1.5)

**12**	0.6 (0.3 to 1.2)	0.7 (0.4 to 1.39	0.7 (0.4 to 1.0)

**13**	0.1 (<0.0 to 0.4)	0.2 (0.03 to 0.5)	0.1 (0.04 to 0.33)

NN-MS Number of non-muscular symptoms; CI Confidence Interval

The mean NN-MS was 3.7 (95% CI 3.6-3.8), 4.0 for women (95% CI 3.8-4.1), and 3.3 for men (95% CI 3.2-3.4).

The mean total number of symptoms (NPS + NN-MS) was 6.0 out of a maximum of 23 (95% CI 5.8-6.2). Women reported 6.7 symptoms (95% CI 6.5-7.0) and men, 5.1 symptoms (95% CI 4.9-5.3) (Table 3). Of the participants, 22.6% (95% CI 21.2-24.0) had 10 symptoms or more (women 27.8% (95% CI 25.8-30.0), men 16.0% (95% CI 14.4-18.3)).

**Table 3:** Prevalence of total symptoms (number of pain sites (NPS) + number of non-muscular symptoms (NN-MS)) by gender

Total symptoms	Men (N = 1455)	Women (N = 1772)	Total (N = 3227)
(NPS+ NN-MS)	% (95% CI)	% (95% CI)	% (95% CI)
**0**	11.5 (9.9 to 13.2)	5.4 (4.4 to 6.5)	8.1 (7.2 to 9.1)

**1**	11.7 (10.2 to 13.5)	8.0 (6.8 to 9.4)	9.7 (8.7 to 10.7)

**2**	9.6 (8.1 to 11.2)	9.4 (8.1 to 10.4)	9.4 (8.5 to 10.5)

**3**	11.2 (9.6 to 12.9)	7.5 (6.4 to 8.8)	9.1 (8.2 to 10.2)

**4**	9.1 (7.8 to 10.7)	8.7 (7.5 to 10.1)	8.9 (7.9 to 9.9)

**5**	8.4 (7.1 to 9.9)	7.8 (6.7 to 9.2)	8.1 (7.2 to 9.1)

**6**	6.9 (5.7 to 8.3)	7.2 (6.1 to 8.5)	7.0 (6.2 to 8.0)

**7**	6.4 (5.3 to 7.8)	7.2 (6.1 to 8.5)	6.8 (6.0 to 7.7)

**8**	5.3 (4.3 to 6.6)	6.1 (5.1 to 7.4)	5.8 (5.0 to 6.6)

**9**	4.4 (3.4 to 5.6)	4.9 (3.9 to 6.0)	4.6 (3.9 to 5.4)

**10**	4.7 (3.7 to 5.9)	5.6 (4.7 to 6.8)	5.2 (4.5 to 6.0)

**11**	3.1 (2.3 to 4.2)	4.7 (3.8 to 5.8)	4.0 (3.3 to 4.7)

**12**	2.2 (1.5 to 3.0)	4.0 (3.1 to 5.0)	3.1 (2.6 to 3.8)

**13**	2.1 (1.5 to 3.0)	3.6 (2.8 to 4.6)	2.9 (2.4 to 3.6)

**14**	1.3 (1.8 to 2.1)	2.1 (1.5 to 2.9)	1.7 (1.3 to 2.3)

**15**	1.8 (0.5 to 1.5)	2.5 (1.8 to 3.3)	1.7 (1.3 to 2.3)

**16**	0.7 (0.4 t 1.3)	1.6 (1.1 to 2.3)	1.2 (0.9 to 1.6)

**17**	0.6 (0.3 to 1.2)	1.6 (1.1 to 2.4)	1.2 (0.9 to 1,6)

**18**	0.1 (0-0.5)	0.9 (0.5 to 1.5)	0.6 (0.4 to 0.9)

**19**	0.4 (0.1 to 0.8)	0.4 (0.2 to 0.8)	0.4 (0.2 to 0.7)

**20**	0.1 (0 to 0.5)	0.2 (0.1 to 0.6)	0.2 (0.1 to 0.4)

**21**	0 (0 to0.2)	0.4 (1.2 to 0.8)	0.2 (0.1 to 0.5)

**22**	0.1 (0 to 0.5)	0.2 (0.1 to 0.6)	0.2 (0.1 to 0.4)

**23**	0 (0 to 0.2)	0 (0 to 0.2)	0 (0 to 0.1)

NPS Number of pain sites; NN-MS Number of non-muscular symptoms; CI Confidence Interval

### The association between NN-MS and NPS

**Figure 1:**
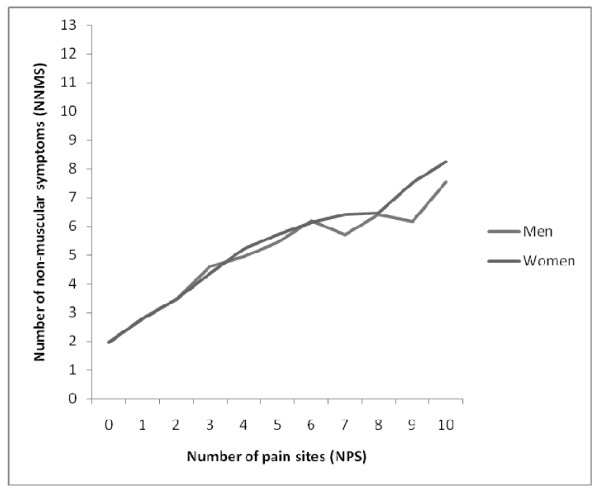
**The association between the number of non-musculoskeletal symptoms (NN-MS) and the number of pain sites (NPS)**. The figure is based on the mean NN-MS score for each NPS-score.

A simple correlation analysis showed a strong association between NN-MS and NPS (r = 0.55), and we found an almost linear relationship (Figure 1). Individuals not reporting any pain sites reported a mean of approximately two non-musculoskeletal symptoms. Respondents with ten pain sites reported a mean of approximately eight non-musculoskeletal symptoms. Results for men and women followed a similar trend.

NN-MS, adjusted for age and gender explained 33.0% of the variance in NPS (R^2 ^= 0.33, *p *< 0.001, Model I), of which the NN-MS -score alone had an explanatory power of 27.1%.

Multicollinearity between symptoms in the analyses was not revealed. The maximum correlation found was 0.56 (between depression and anxiety), and the collinearity indicators Tolerance and Variance Inflation Factors (VIF) were well within the limits of concern for all symptoms involved in the analysis. Depression had the lowest tolerance (= 0.59) and the highest VIF (= 1.69).

When modelling NPS as a function of the 13 individual non-musculoskeletal symptoms, age and gender (Model II), the explanatory power was 33.8% (R^2 ^= 0.338, *p *< 0.001). The 13 non-musculoskeletal symptoms alone explained 28.2% of the variance in NPS. The individual symptoms explained between 2.4% (eczema) and 12.0% (dizziness) of the variance in NPS. Dizziness, breathing difficulties and chest pain were the symptoms that gave the greatest increase in NPS with β = 1.86, 1.78, and 1.76, respectively (Table 4) The β-values indicate the increase in NPS-score if the symptom analysed is present vs. not present.

**Figure 2:**
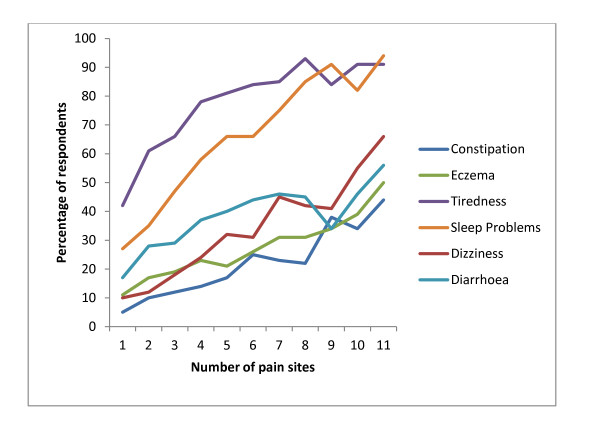
**The number of pain sites (NPS) in relation to the three non-musculoskeletal symptoms with the highest explanatory power in NPS (dizziness, sleep problems, and tiredness) and the three non-musculoskeletal symptoms with the lowest explanatory power in NPS (diarrhoea, constipation, and eczema)**. The figure is based on the mean score of the symptoms for each NPS-score.

We found that the prevalence of each non-musculoskeletal symptom was almost linearly related to NPS (Figure 2). The lines for individual non-musculoskeletal symptoms had similar slopes but different intercepts. Tiredness had the highest intercept; approximately 40% reported this symptom at zero pain sites, whereas chest pain had the lowest intercept, with approximately 5% reporting this symptom at zero pain sites.

Finally, we entered the individual non-musculoskeletal symptoms in the model, controlling for the remaining 12 non-musculoskeletal symptoms in addition to age and gender (Model III, Table 4). The explanatory power of the individual symptoms was greatly reduced compared to Model II, explaining from 0.1% to 1.9% of the variance in NPS. The symptoms giving the greatest increase in NPS were dizziness and sleep problems (β = 0.78 and 0.74 respectively).

**Table 4:** Linear regression analyses of the association between number of pain sites (NPS) and individual non-muscular symptoms

	Model II^1^				Model III^2^			
**Symptom**	**β**	**(95% CI)**	**R^2^**	***p*-value**	**β**	**(95% CI)**	**R^2^**	***p*-value**

**Palpitations**	1.33	(1.14-1.52)	0.051	<0.001	0.22	(0.03-0.40)	0.001	0.020

**Chest pain**	1.76	(1.56-1.96)	0.078	<0.001	0.51	(0.31-0.72)	0.005	<0.001

**Breathing difficulties**	1.78	(1.58-1.98)	0.081	<0.001	0.53	(0.33-0.73)	0.005	<0.001

**Heart burn**	1.08	(0.91-1.25)	0.044	<0.001	0.20	(0.05-0.36)	0.001	0.12

**Stomach discomfort**	1.67	(1.49-1.85)	0.085	<0.001	0.51	(0.33-0.70)	0.006	<0.001

**Diarrhea**	1.10	(0.93-1.27)	0.043	<0.001	0.35	(0.20-0.51)	0.004	<0.001

**Constipation**	1.34	(1.10-1.57)	0.035	<0.001	0.43	(0.22-0.64)	0.003	<0.001

**Eczema**	0.93	(0.73-1.13)	0.024	<0.001	0.40	(0.22-0.57)	0.004	<0.001

**Tiredness**	1.52	(1.36-1.68)	0.093	<0.001	0.43	(0.28-0.59)	0.006	<0.001

**Dizziness**	1.86	(1.69-2.03)	0.120	<0.001	0.78	(0.61-0.95)	0.017	<0.001

**Anxiety**	1.63	(1.45-1.82)	0.080	<0.001	0.26	(0.05-0.47)	0.001	0.14

**Depression**	1.51	(1.35-1.68)	0.089	<0.001	0.31	(0.12-0.49)	0.002	0.001

**Sleep problems**	1.57	(1.41-1.72)	0.104	<0.001	0.74	(0.59-0.89)	0.019	<0.001

β unstandardised regression coefficient; R^2 ^multiple correlation coefficient

^1 ^Model II: Dependent variable: Number of pain sites (NPS). Independent variable: Individual non-muscular symptoms while controlling for age and gender

^2 ^Model III: Dependent variable: Number of pain sites (NPS). Independent variable: individual non-muscular symptoms while controlling for the remaining 12 non-muscular symptoms in addition to age and gender

In the questionnaire, non-musculoskeletal symptoms were graded into levels of severity. Including only symptoms graded as affecting the respondent to "some" and "severe" degree, the explanatory power was reduced to 28.9%. Including "severe" symptoms only, NN-MS explained 17.5% of the variance in NPS.

Sensitivity analyses, where all analyses were performed on non-imputated data, showed that the imputation procedures had a tendency to weaken rather than strengthen the associations presented in our results (data not shown).

## Discussion and conclusions

### Key findings

We have found that a substantial part of the population report a great number of symptoms. There is a strong, almost linear relationship between the number of non-musculoskeletal symptoms (NN-MS) and the number of pain sites (NPS). Similarly, the prevalence of the individual non-musculoskeletal symptoms increased with increasing NPS.

NN-MS explained 27.1% of the variance in NPS. In comparison, a model comprising the 13 individual non-musculoskeletal symptoms explained 28.2% of the variance in NPS. The individual symptoms explained between 2.4% and 12.0% of the variance in NPS, when controlling for age and gender.

### Methodological considerations

#### Study sample and design

This study is population-based and has a relatively large sample size. However, some precautions should be considered when interpreting the results due to the modest response rate. Some studies have pointed out that responders tend to have better health than drop-outs ("the healthy volunteer effect"), [26-28]. On the other hand, it might be that individuals experiencing symptoms asked for in the study found it more interesting and, therefore, decided to participate [29]. In addition, another Norwegian population survey found no differences in lifestyle factors between responders and non-responders [30]. We have no data on symptoms experienced by non-responders and are, therefore, unable to draw conclusions about the representability of participants' reporting of symptoms. Higher response rates in the groups reporting most symptoms (i.e. women and middle-aged) can indicate that the effect of interest in the study have been more important than the healthy volunteer effect, and this might have caused some overestimation of the prevalence figures.

Selection bias might influence prevalence numbers reported in this paper, but it is unlikely that the strong association between non-musculoskeletal symptoms and NPS is affected in such a way that it will alter our conclusions.

One limitation of our cross-sectional design is that it only allows evaluation of the association between variables and makes us unable to infer any cause-effect relationship.

### Assessment of symptoms

Given the complex and subjective nature of symptoms, self-report methods are the best, if not the only possible approach [31]. The quality of the data is dependent on participants' honesty and willingness to participate [32].

SNQ has been widely used in different versions, and is considered to be well-validated and reliable [23].In the Ullensaker study, we added a tenth body region (head) to the original nine pain regions. The SNQ does not ask directly whether the pain or discomfort experienced is musculoskeletal in origin, although this may be an implicit assumption.

We have modified the original symptom reporting instrument SHC by omitting musculoskeletal-related symptoms. The questions on pain symptoms asked for were dichotomous, and included "pain and discomfort"; hence we included individuals even "a little" bothered by the non-musculoskeletal symptoms.

By using these instruments, we have a mis-match between the 7-day time window in SNQ and the 30-day window in SHC. The different time frames might influence the association between the two variables, but this will probably weaken rather than strengthen the association.

Some individuals might have a tendency to report any symptoms, whereas others might consider similar symptoms to be insignificant and, hence, not report them. The number of symptoms reported and the strong association between NN-MS and NPS could, therefore, simply be a result of reporting behaviour. It could be argued that personality traits may influence reporting patterns, but such traits would at the same time influence the consequences of the reported symptoms, such as health care use, sickness absence and disability pensioning. In an earlier paper from the Ullensaker Study, it was found that NPS is an important predictor for future disability pensioning, demonstrating that NPS has a strong predictive validity.

### Consideration of statistical methods

There are methodological issues concerning dichotomisation that need to be considered. In order to be able to count the number of symptoms, we needed a cut-off for what was to be considered "symptom reported" and "symptom not reported". In line with the previous papers in the Ullensaker study, we wanted to include all levels of intensity for each symptom. Dichotomisation might in itself influence the association between data, introducing potential information bias. Analyses moving the cut-off for dichotomisation to include only symptoms affecting respondents to "some" or "severe" degree, gave reduced explanatory power in NPS. Introduction of bias might also occur for the imputation methods used, although our sensitivity analyses indicated that our imputations were conservative.

### Discussion of results

People report a great number of symptoms when directly asked for them in surveys [2,33], symptoms that do not necessarily reflect underlying disease. In our study, 22.6% reported 10 or more symptoms out of 23. Persons with medically unexplained symptoms (MUS) usually report a number of symptoms [34], and most of them would accordingly be in the groups reporting most symptoms.

We have found a linear association between NN-MS and NPS (Figure 1). If we are to regard the number of symptoms reported as an indicator of health or well-being, there seems to be a continuum of this state in our population. Although our results are not unexpected, it is important to have the associations demonstrated in population data. Even individual non-musculoskeletal symptoms, although clinically varied in nature, are related to NPS in an almost linear fashion. Knowing the respondent's answers to each of the 13 individual symptoms adds little in explaining NPS compared to simply knowing the number.

The linear relationship between the number of musculoskeletal and non-musculoskeletal symptoms might indicate an internal association in symptom reporting between symptoms, where, for example, individuals experiencing pain will be prone to depression and sleep problems. On the other hand, the strong linear relationship between the number of musculoskeletal and non-musculoskeletal symptoms supports the position that the symptoms might share some common characteristics and even common underlying causal factors. Hence, symptom reporting could be looked upon as a phenomenon in itself, independent of diagnoses.

It is well known that some individuals with musculoskeletal pain also report a variety of other symptoms. Non-musculoskeletal symptoms were even reintroduced in the 2010 preliminary criteria for fibromyalgia by the American College of Rheumatology [19,35], after having been abandoned in the 1990 version of the criteria, not because they were irrelevant, but because they were judged not to contribute to the specificity of the diagnosis of fibromyalgia [36].

In an earlier study on subjective health complaints in the general population, a main finding was that there were no sharp or obvious limits separating "normal" and endurable pain and complaints and complaints that were in need of professional help and might get labelled as a specific diagnosis or syndrome [3]. In the contemporary discussion of symptoms, most focus has been on medically unexplained symptoms and multisymptom reporting [10,12,33,37-40]. Some studies focusing on medically unexplained symptoms (MUS) use a definition merely as a function of the number of symptoms the individual reports. Thus, the more symptoms you have, the more likely it is that they are medically unexplained. For example, the validated questionnaire PHQ-15 assesses 15 symptoms or symptom clusters that "account for more than 90% of the physical complaints reported in the outpatient setting" [2]. Kroenke et al. state that the PHQ cannot distinguish between medically explained and medically unexplained symptoms, but emphasize that "the total symptom counts (including medically unexplained and explained) are predictive of somatoform disorders and correlate strongly with psychological distress, functional impairment and health care utilization"[41-43]. The different medically unexplained syndromes overlap, and the diagnoses are based entirely, or in part, on symptoms widely reported in the general population.

NPS has revealed itself to have strong predictive validity, and NN-MS seems to be another informative dimension in describing the pattern of symptom reporting in the general population. Hence, the total burden of symptoms as indicated by the number of symptoms reported might be an interesting generic indicator of an individual's health or well-being, as well as his/her present and future functioning, and may be worth further investigations.

In our approach, based on the history of our research, we have distinguished between musculoskeletal and non-musculoskeletal symptoms. Other studies have distinguished between somatic and mental symptoms. Both epidemiological and clinical research on symptom reporting should avoid such distinctions and instead include all relevant symptoms.

Symptom reporting is the main entrance into the health care system. One implication of our findings is that health professionals in general and GPs in particular might become more aware of the "normal" range of symptom reporting in the population and, thereby, be better equipped to decide which individuals should undergo supplementary medical investigations. Research on symptom reporting might also be an alternative pathway to describe and possibly understand the medically unexplained multisymptom conditions.

## Competing interests

The authors declare that they have no competing interests.

## Authors' contributions

HTM authored the manuscript and performed the statistical analysis. DB, MK, JS, BN and CI all helped draft the manuscript and revise the manuscript critically for important intellectual and made contributions to the interpretations of data. All authors made contributions to the interpretation of data and revise the manuscript critically for intellectual content. DB, BN and CI have made substantial contributions to conception and acquisition of data. ID and YK have made contributions to analysing data. All authors read and approved the final manuscript.

## Pre-publication history

The pre-publication history for this paper can be accessed here:

http://www.biomedcentral.com/1471-2474/12/285/prepub
